# Characterization of Neuronal Populations in the Human Trigeminal Ganglion and Their Association with Latent Herpes Simplex Virus-1 Infection

**DOI:** 10.1371/journal.pone.0083603

**Published:** 2013-12-19

**Authors:** Sarah E. Flowerdew, Desiree Wick, Susanne Himmelein, Anja K. E. Horn, Inga Sinicina, Michael Strupp, Thomas Brandt, Diethilde Theil, Katharina Hüfner

**Affiliations:** 1 Department of Neurology, Klinikum Grosshadern, Ludwig-Maximilians University, Munich, Germany; 2 German Center for Vertigo and Balance Disorders, Ludwig-Maximilians University, Munich, Germany; 3 Institute of Anatomy, Department I, Ludwig-Maximilians University, Munich, Germany; 4 Department of Legal Medicine, Ludwig-Maximilians University, Munich, Germany; 5 Institute for Clinical Neurosciences, Klinikum Grosshadern, Ludwig-Maximilians University, Munich, Germany; University of Cambridge, United Kingdom

## Abstract

Following primary infection Herpes simplex virus-1 (HSV-1) establishes lifelong latency in the neurons of human sensory ganglia. Upon reactivation HSV-1 can cause neurological diseases such as facial palsy, vestibular neuritis or encephalitis. Certain populations of sensory neurons have been shown to be more susceptible to latent infection in the animal model, but this has not been addressed in human tissue. In the present study, trigeminal ganglion (TG) neurons expressing six neuronal marker proteins were characterized, based on staining with antibodies against the GDNF family ligand receptor Ret, the high-affinity nerve growth factor receptor TrkA, neuronal nitric oxide synthase (nNOS), the antibody RT97 against 200kDa neurofilament, calcitonin gene-related peptide and peripherin. The frequencies of marker-positive neurons and their average neuronal sizes were assessed, with TrkA-positive (61.82%) neurons being the most abundant, and Ret-positive (26.93%) the least prevalent. Neurons positive with the antibody RT97 (1253 µm^2^) were the largest, and those stained against peripherin (884 µm^2^) were the smallest. Dual immunofluorescence revealed at least a 4.5% overlap for every tested marker combination, with overlap for the combinations TrkA/Ret, TrkA/RT97 and Ret/nNOS lower, and the overlap between Ret/CGRP being higher than would be expected by chance. With respect to latent HSV-1 infection, latency associated transcripts (LAT) were detected using in situ hybridization (ISH) in neurons expressing each of the marker proteins. In contrast to the mouse model, co-localization with neuronal markers Ret or CGRP mirrored the magnitude of these neuron populations, whereas for the other four neuronal markers fewer marker-positive cells were also LAT-ISH+. Ret and CGRP are both known to label neurons related to pain signaling.

## Introduction

Herpes simplex virus-1 (HSV-1) is a human neurotropic DNA virus of the herpesviridae family [1]. After an initial oral infection, the virus can travel along axons innervating the affected region, to reach the trigeminal ganglia (TG) where it establishes a lifelong latency. The sensory neurons of the TG are the principal site for HSV-1 latency in humans, although the vestibular, geniculate, spiral, and sacral ganglia can also harbor latent virus as summarized in [2], [3]. During latency HSV-1 viral activity is restricted, with only the latency associated transcript (LAT) being abundantly expressed [4]. LAT is engaged in establishing latency [5], [6], in facilitating the process of reactivation [7], [8], and at the same time promoting neuronal survival after HSV-1 infection by reducing apoptosis [9]. Hence, it is reasonable to consider LAT a surrogate marker of HSV-1 latency. Reactivation can occur spontaneously or be induced by various stimuli, leading to diseases including herpes labialis, facial palsy, vestibular neuritis or encephalitis [10], [11]. The mechanisms underlying establishment and maintenance of latency, as well as viral reactivation, are still under investigation. Several viral factors have been identified which may play a role [2], [11], [12], but host factors which influence latency and reactivation are less well understood: the host´s immune system definitely plays an important role [13]–[16], but data from the animal model suggest that neuronal characteristics might also be important.

Sensory neurons in the TG form a diverse population of cells that can be classified according to cellular morphology, neurochemistry and functional characteristics [17], [18]. Classically, studies of the peripheral nervous system have subdivided the neurons into three major classes on the basis of their sizes: A (also known as large light), B (also known as small dark) and C [19]. Neurochemical classification of neurons is based on their protein expression profile, including the presence of growth factor receptors, neuropeptides and neurotransmitters [20], but a full neurochemical classification of neurons in the human TG has not been performed.

Different neuronal populations have been shown to display variable susceptibilities for HSV-1 infection in the mouse model. Although all TG neuronal populations in the mouse model are capable of supporting an HSV-1 infection, neurons positive with the monoclonal antibody KH10 are more permissive for a productive infection [21], while those expressing the globoseries glycoconjugate stage specific embryonic antigen 3 (SSEA3) are productively infected less than might be expected from the magnitude of their neuronal population [22]. Similarly, during latent infections in the mouse model, trigeminal and dorsal root neuronal populations reactive for the monoclonal antibody A5 are more susceptible [21]–[23] than neurons positive for KH10 [18].

In the current study, we characterized the neuronal composition of the human TG using six different marker proteins: the GDNF family ligand receptor Ret, the high-affinity nerve growth factor receptor TrkA, neuronal nitric oxide synthase (nNOS), the antibody RT97 against 200 kDa neurofilament, calcitonin gene-related peptide (CGRP) and peripherin (for details see Table 1). Secondly, we assessed the susceptibility of neurons expressing these markers towards latent HSV-1 infection via the expression of HSV-1 LAT using in situ hybridization (ISH).

**Table 1:** Characteristics and frequency of neuronal markers analyzed.

	Antigen recognized by antibody	Expected cellular localization^a^	Proposed characteristics of labeled neurons	Data from animal models	% labeled neurons (number of counted neurons (present study))
Ret	GDNF-family ligand receptor tyrosine kinase	Membrane protein, also seen as cytoplasmic and Golgi ^hn^ [34]	Small diameter neurons, especially nociceptors [51]	Labels neurons equivalent to KH10 antibody in mouse [23]	26.93 (2306)
TrkA	High-affinity nerve growth factor receptor tyrosine kinase	Cytoplasmic and Golgi ^hn^ [52] Plasma membrane ^ho^ [53]	Small diameter neurons, especially nociceptors [51]	Labels neurons equivalent to A5 antibody in mouse [23]	61.82 (2499)
nNOS	Neuronal nitric oxide synthase	Cytoplasmic ^hn^ [54]	Prevalence in ophthalmic branch of TG [20]	NO can inhibit HSV-1 replication [21]	33.13 (2792)
RT97	Phosphorylated 200kDa neurofilament	Cytoplasmic ^an^ [19], [55]	Larger A-type neurons [45]		39.26 (2127)
CGRP	Calcitonin gene-related peptide, a vasodilator and pain mediator	Cytoplasmic ^hn^ [56]	Smaller sized neurons [20]	Found throughout the TG of mice [20], and innervate the gingiva in rats [33]	27.16 (2003)
Peripherin	Type III intermediate filament	Cytoplasmic ^hn^ [57]	Neurons of the peripheral nervous system and small nerve fibers of the sensory ganglia [58]	Upregulated in response to nerve injury and proinflammatory cytokines [59]	40.87 (2109)

a: References are marked according to whether the experiments were performed in humans (h) or animals (a), and neurons (n) or other cell types (o).

## Materials and Methods

### Ethics statement

The use of autopsy samples for the present study was approved by the Ethics Committee of the Medical Faculty of the Ludwig-Maximilians University, Munich, Germany (project number: 017-11). The tissue samples were collected from the cadavers as part of the routine autopsy procedure and were irreversibly anonymized, so that according to the national ethics guidelines obtaining consent is neither possible nor required. The Ethics Committee approved this consent procedure.

### Human tissue samples

Fourteen human trigeminal ganglia (5 LAT-negative, 9 LAT-positive; Table 2) from 14 individuals were obtained at autopsy at Ludwig-Maximilians University (Munich, Germany). LAT negativity or positivity was determined using ISH at different levels within each ganglion (see protocol below) and RT-PCR analysis [24] which gave identical results. All LAT+ samples were also positive using nested HSV-1 DNA PCR [25], while all LAT- samples were negative using this method. Nested HSV-2 DNA PCR [26] was negative in all samples. Samples 1–10 (Table 2) were used for dual LAT ISH and marker protein staining, and samples 11–14 (Table 2) for double marker protein fluorescence staining. The average age at death was 38yr±8 for the LAT– and 55yr±5 for the LAT+ samples, and the average post-mortem delay was 15hr±2.4 for LAT– and 23hr±2.3 for the LAT+ samples. No donors had evidence of active herpesvirus infection at death. Ganglia were embedded directly in Jung Tissue Freezing Medium (Leica Microsystems, Nussloch, Germany) and stored at –80°C for further use.

**Table 2:** Information on trigeminal ganglion donors.

ID	LAT status	Sex*^a^*	Age at death (years)	Cause of death
1	–	M	35	Cardiac
2	–	M	29	Drug overdose
3	–	F	36	Cardiac
4	–	F	20	Cardiac
5	–	F	70	Cardiac
6	+	F	78	Cardiac
7	+	M	57	Trauma
8	+	F	41	Cranial aneurism
9	+	M	69	Cardiac
10	+	M	47	Cardiac
11	+	M	71	Cardiac
12	+	M	44	Trauma
13	+	M	40	Cardiac
14	+	M	50	Trauma

*a*: M = Male, F = Female.

### In situ analyses

It is still a matter of debate whether LAT can be visualized in all latently infected neurons using ISH or if some neurons do not express LAT to a level which allows for detection using this method [27], [28]. We thus refer to the neurons in this manuscript as “LAT-ISH+” or “LAT-ISH– “ to indicate this fact. In situ hybridization was performed in combination with immunohistochemistry as described previously [27]. In brief, defrosted tissue sections were paraformaldehyde-fixed, then endogenous peroxidase-blocked using 1.5% H_2_O_2_ in methanol, acetylated with 0.25% acetic anhydride in triethanolamine, then prehybridized for 1hr in PHO buffer (1xDenhard’s solution, 0.1 µg/ml herring sperm DNA, 5 mg/ml sodium pyrophosphate, 5 mM Tris-HCl, in 4x sodium citrate buffer SSC). Samples were incubated overnight at 37°C with 4 ng/µl of an oligonucleotide probe against HSV-1 LAT (not recognizing HSV-2 LAT), labeled with a 3′-DIG tag (5′-CAT AGA GAG CCA GGC ACA AAA ACA C-3′; nucleotides 119.783 – 119.759; Eurofins MWG Operon, Ebersberg, Germany). After further washing and blocking, samples were incubated for 2 hours with an anti-DIG-AP conjugated antibody (Roche Diagnostics, Mannheim, Germany), and reactivity was visualized using NBT/BCIP (Roche Diagnostics). Samples were again fixed in paraformaldehyde, then incubated overnight with primary antibodies at pre-determined optimal dilutions, before this reactivity was visualized using biotin-conjugated secondary antibodies (Dako, Hamburg, Germany; Abcam, Cambridge, UK), HRP-conjugated streptavidin (BioLegend, Fell, Germany), and 3-3′-diaminobenzidine (DAB; Dako). Samples were counterstained with haematoxylin to help visualize cells and nuclei.

Primary antibodies used in this study: mouse monoclonal to Ret (Ret01), ab1840, Abcam, Cambridge, UK, used at 1∶10; goat polyclonal to TrkA, AF175, R&D Systems, Wiesbaden, Germany, used at 1∶50; rabbit polyclonal to nNOS, AB5380, Merck Millipore, Molsheim, France, used at 1∶2000; mouse monoclonal to NEFH (RT97), MA1-34345, Thermo Scientific/Pierce Biotechnology, Rockford, IL, USA, used at 1∶100; sheep polyclonal to CGRP, CA1137, Enzo Life Sciences, Lörrach, Germany, used at 1∶1500; rabbit polyclonal to peripherin, ab4666, Abcam, Cambridge, UK, used at 1∶750. Controls without primary or secondary antibodies as well as with isotype control antibodies were performed (see Figures S1 and S2). No mouse ascites derived antibodies were used in the current study [29]. All experiments were performed in duplicate or triplicate on non-consecutive sections. Staining was visualized using an Olympus BX41 microscope and pictures were taken with an Olympus C4040 Zoom digital camera. Images were adjusted in CorelDraw to reflect the brightness and contrast as seen through the microscope.

### Fluorescence immunohistochemistry for dual visualization

For fluorescence IHC experiments, sections from LAT+ donors were paraformaldehyde fixed, blocked in 2% bovine serum albumin (BSA, Sigma Aldrich, Steinheim, Germany), then exposed to the first primary antibody for 2 hours at room temperature. Antibodies were used at the same concentrations as given above. After washing in PBS, slides were incubated with an appropriate fluorophore-conjugated secondary antibody, as listed below, for 30 minutes. After further washing and blocking with BSA, slides were then incubated with the second primary antibody overnight, at 4°C, then washed again, and incubated with a secondary antibody of alternative color. After washing in both PBS and 75% ethanol, slides were incubated in a solution of 1% Sudan Black (Sigma Aldrich) in 70% ethanol for 10 minutes to reduce lipofuscin autofluorescence. Slides were then washed in 75% ethanol, before being mounted with Fluoro-Gel II, with DAPI (Science Services, Munich, Germany). Controls omitting primary or secondary antibodies as well as using isotype control antibodies were performed (see Figure S3).

All secondary antibodies were purchased from Life Technologies Molecular Probes (Darmstadt, Germany), and used at a 1∶200 dilution. These included AlexaFluor568 conjugated donkey anti-goat, donkey anti-rabbit and donkey anti-mouse, and AlexaFluor488 conjugated goat anti-mouse and goat anti-rabbit. All experiments were performed in duplicate. Slides were visualized using an All-in-one Type Fluorescence Microscope (BZ-8100E, Keyence) and images were obtained with an integrated camera and software. Images were adjusted in CorelDraw to reflect the brightness and contrast as seen through the microscope.

### Neuron assessment and measurement

Following staining, neurons were assessed and counted based on their staining pattern. Only neurons with a visible nucleus were counted to ensure potential LAT staining was not missed. Neurons were assigned as singly or doubly positive or negative.

For size analysis, only neurons with a nucleus were measured, as this suggested that the neuron was cut through a central plane. Additionally, neurons were only included in the analysis where the surrounding tissue was intact to confirm that the neuron had not shrunk post-mortem or during the experimental procedure. Due to these strict inclusion criteria many neurons could not be assessed for the current study, and thus a bias towards more “robust” neurons cannot be excluded. The cross-sectional area of neurons inµm^2^ was measured using Image J (Rasband, W.S., ImageJ, U. S. National Institutes of Health, Bethesda, Maryland, USA, http://imagej.nih.gov/ij/, 1997–2012.). The measurements were calibrated by imaging a calibration grid using the same microscope and camera settings, and calculating the distance per pixel.

### Statistical analysis

Statistical analyses were performed in Microsoft Excel and SPSS, p<0.05 was regarded as significant for all analyses. For data with a Gaussian distribution mean ± SEM are given, for those with non-Gaussian distribution, values for the median, 25^th^ and 75^th^ percentiles are presented. Differences in the percentage of Marker+ neurons between LAT-ISH+ and LAT-ISH– samples, and between DAB marker staining and fluorescence staining, were calculated using a two-tailed Mann-Whitney U test. Correlations between the age or post-mortem delay of the samples, and the percentage of positive staining were calculated using the Spearman Correlation coefficient and 2-tailed T-test. Differences in the sizes between Marker+ and Marker– neurons from the same experiments were calculated using a Mann-Whitney U-test. For analysis of the co-localization of two marker proteins, the Pearson Chi-squared test was used to calculate p-values. For analysis of the co-localization of LAT with marker proteins, the two-tailed Fisher exact Chi-squared test was used to calculate p-values, due to the low incidence of LAT-ISH+ neurons.

## Results

### Distribution of neuronal marker proteins in TG neurons

**Figure 1:**
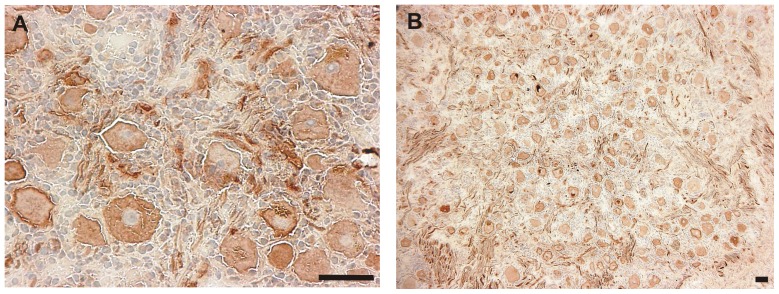
Labeling of a TG with a marker mix of all markers used in the current study (Ret, TrkA, nNOS, RT97, CGRP and peripherin). The micrograph on the left (A) was taken at 400x magnification for a more detailed view, that on the right (B) at 100x for an overview. Marker proteins are labeled with DAB (brown) resulting in complete labeling of all detected neurons. The tissue was counterstained with haematoxylin. Scale bars represent 50 µm in all cases.

Sections from 5 LAT+ donors and 5 LAT− donors (donors 1-10, Table 2) were stained immunohistochemically for each of six neuronal marker proteins (Table 1). Neurons positive for the high-affinity growth factor receptor TrkA were most abundant with 61.82% of all assessed neurons staining positive, peripherin was detected in 40.87%, the neurofilament antibody RT97 in 39.26%, the neuronal nitric oxide synthase nNOS in 33.13%, calcitonin gene-related peptide (CGRP) in 27.16% and the GDNF family ligand receptor Ret in 26.93% of all neurons. The percentage of Marker+ neurons was compared between the LAT+ and LAT− donors. No differences in frequency between LAT+ and LAT− donors were found for any neuronal marker proteins (p>0.05 in all cases, Mann-Whitney U test, 2-tailed). Correlations between the number of Marker+ neurons and either the age of the donor, or the post-mortem delay between death and autopsy, were also evaluated. No correlations were seen, except for a negative correlation between age and the percentage of neurons positive for CGRP (Spearman correlation coefficient –0.73, T-test p = 0.027; data not shown). Experiments performed using all six marker protein antibodies gave complete coverage of all TG neurons (Figure 1).

### Size of differential neuronal populations in TG

**Figure 2:**
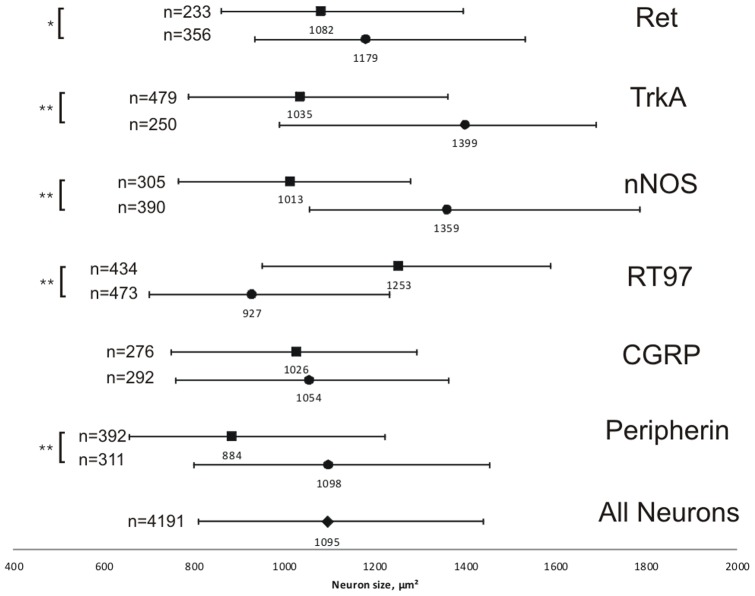
Median cross-sectional areas of cells labeled with different marker antibodies. Central values represent the median values inµm^2^, whilst the bars indicate the 25th–75th percentile ranges. Filled squares  =  marker positive neurons, filled circles  =  marker negative neurons from the same experiment. n represents the number of counted neurons. Asterisks represent significant differences (*  =  p<0.01, **  =  p<0.001, Mann-Whitney U-test).

To further characterize the neurons expressing each marker protein, the cross-sectional area of neurons was measured for both neuronal Marker+ and Marker− cells from the same experiments (Figure 2). The median for all neurons, both labeled and unlabeled from all experiments, was 1095 µm^2^. In the case of Ret, TrkA, nNOS and peripherin, those neurons expressing the protein were significantly smaller than those negative for staining on the same slides (p = 0.013 (Ret) and p<0.001 (TrkA, nNOS, peripherin), Mann-Whitney U test, 2-tailed), whilst for RT97, positive neurons were significantly larger (p<0.001 Mann-Whitney U test, 2-tailed). Cells stained with antibody against CGRP showed no significant difference in cross-sectional area compared with negative neurons. All of the markers stained neurons from a wide range of sizes with a large overlap between the different neuronal marker proteins (Figure 2).

### Co-localization of marker proteins in neurons

**Figure 3:**
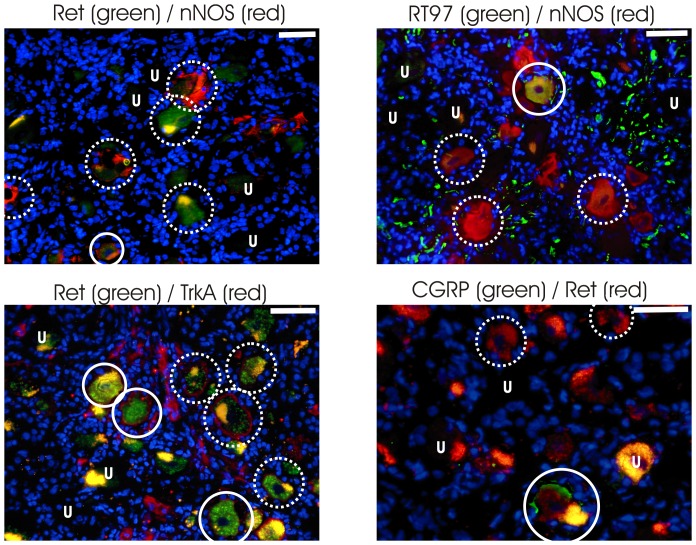
Fluorescence co-labeling of marker combinations . The markers are labeled with Alexa568 (red), Alexa488 (green) and DAPI (blue). Marker combinations are indicated above each pictogram. Dotted-line circles indicate examples of single labeled neurons, solid-line circles indicate examples of double labeled neurons. The letter “U” indicates examples of unlabeled neurons, some with lipofuscin. Scale bars represent 50 µm.

The high percentage of neurons expressing each of the six marker proteins investigated suggested that there was some overlap in the expression of these proteins. To investigate this co-expression, we performed dual immunofluorescence experiments for a subset of antibody combinations in LAT+ donors (samples 11-14, Table 2, Figure 3). The percentage of cells labeled with TrkA and nNOS using fluorescence secondary antibodies was significantly lower (p<0.05, Mann-Whitney U test, 2-tailed) compared to DAB staining, possibly reflecting inter-donor variability or increased sensitivity due to the extra streptavidin amplification in the DAB protocol.

With all of the tested combinations of antibodies, a subset of neurons was observed to be double labeled (Marker 1+/Marker 2+ in Table 3). The greatest overlap was between TrkA and RT97 (20.13%), whilst the least was between Ret and nNOS (4.5%). These results were analyzed using a Chi-squared test, to confirm if this overlap reflected the distribution of the individual markers in the population of neurons as a whole. The co-localizations of TrkA/nNOS, and nNOS/RT97 were as expected by chance, based on the frequency of these markers in the population. However, the Chi-squared results for TrkA/Ret, TrkA/RT97, Ret/nNOS and Ret/CGRP were all significant (p<0.05, see Table 3 for exact values and Table S1 for median and interquartile ranges). Comparison of the expected and observed frequencies shows that the co-localization of Ret/CGRP was more frequent than expected by chance, while it was less than expected by chance for the combinations TrkA/Ret, TrkA/RT97 and Ret/nNOS.

**Table 3:** Co-localization of neuronal marker proteins using immunofluorescence^a,b^.

Marker 1	Marker 2	Marker 1+/ Marker 2+	Marker 1+/ Marker 2–	Marker 1–/ Marker 2+	Marker 1–/ Marker 2–	p^c^
Ret	nNOS	4.5%	23%	16.63%	55.88%	0.042*
Ret	CGRP	15.75%	19.13%	16.88%	48.25%	<0.01*
TrkA	Ret	8.38%	34.75%	15%	41.88%	0.021*
TrkA	nNOS	10%	35.88%	14.5%	39.63%	0.102
TrkA	RT97	20.13%	25.25%	19.63%	35%	0.015*
nNOS	RT97	9.25%	17.75%	27%	46%	0.476

a: see Supplementary Table S4 for median and interquartile ranges

b: 800 neurons were counted per marker pair.

c: p-value as calculated by the Pearson Chi-squared test, with p<0.05 taken as significant, shown by an asterisk.

### Frequency and size of LAT-ISH+ neurons in LAT+ donors

Using samples from 5 LAT+ donors (samples 6–10 in Table 2), individual neurons were assessed to be LAT-ISH+ or LAT-ISH−. Overall, 4.72%±0.39 (mean ± SEM) of neurons were LAT-ISH+, indicating latent HSV-1 infection. To further characterize these infected neurons, the cross-sectional area of LAT-ISH+ neurons was measured and compared to LAT-ISH- neurons from the same donors. The median cross-sectional area of LAT-ISH+ neurons was 1106 µm^2^ (821 µm^2^–1439 µm^2^; 25^th^ percentile - 75th percentile) and of LAT-ISH- neurons was 1070 µm^2^ (786 µm^2^–1429 µm^2^). There was no significant difference between the sizes of LAT-ISH+ and LAT-ISH– neurons (p = 0.502, Mann-Whitney U test, 2-tailed). The median cross-sectional area of 1106 µm^2^ for LAT-ISH+ neurons is closest in size to the median of Ret-positive neurons (Figure 2).

### Co-localization of LAT and neuronal markers in LAT+ ganglia

**Figure 4:**
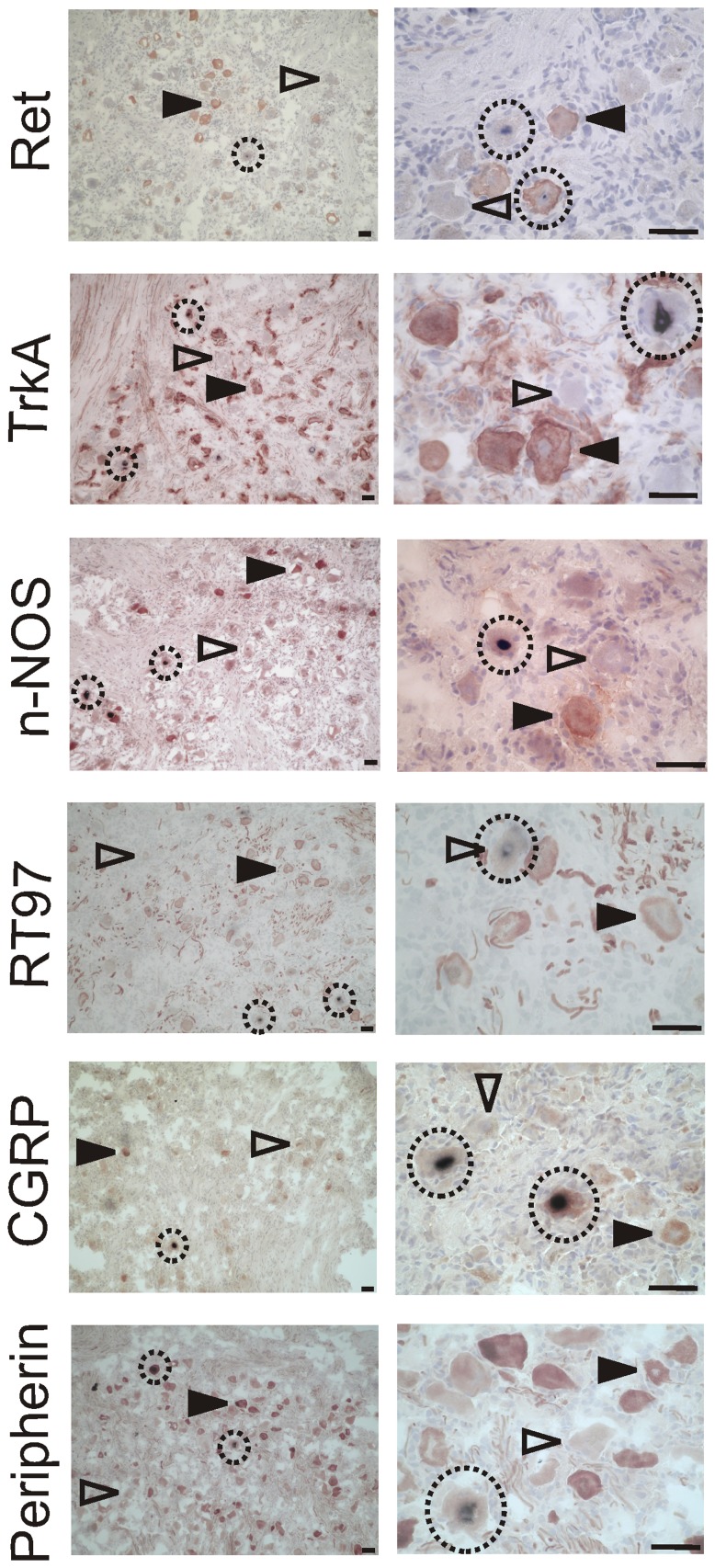
Detection of HSV-1 LAT in neurons expressing different maker proteins in the human TG. The micrographs in the left column are taken at 100x magnification for an overview, those in the right column at 400x for a more detailed view. The name of the marker used is indicated in each row. Marker proteins are labeled with DAB (brown), LAT using NBT (purple), and the tissue was counterstained with haematoxylin. Open arrowheads indicate marker negative cells, closed arrowheads marker positive cells. LAT positive cells are circled. Scale bars represent 50 µm in all cases.

Slides from LAT+ donors (samples 6-10, Table 2) were co-stained for LAT and one of each of the six markers investigated in this study, and assigned to one of four categories. These categories were: LAT-ISH+, and either Marker+ or Marker–, or LAT-ISH– and either Marker+ or Marker– (Table 4, Figure 4 and Table S2 for median and interquartile ranges).

**Table 4:** Percentage of neurons labeled for neuronal markers and LAT^a^.

Marker	LAT+ / Marker+	LAT+ / Marker–	LAT– / Marker+	LAT– / Marker–	No. counted neurons	P^b^
Ret	0.95%	3.39%	25.50%	70.15%	1149	0.516
TrkA	1.87%	3.82%	54.91%	39.40%	1231	<0.001*
nNOS	0.98%	4.29%	32.33%	62.40%	1423	0.0054*
RT97	0.63%	3.59%	39.37%	56.41%	1115	<0.001*
CGRP	0.6%	3%	24.8%	71.6%	1000	0.249
Peripherin	0.73%	3.09%	40.20%	55.99%	1102	0.0035*

a: see Table S2 for median and interquartile ranges.

b: p-value calculated using Fisher’s exact Chi-squared test, with p<0.05 taken as significant, shown by an asterisk.

The distribution of LAT-ISH+ neurons with and without the marker protein was compared to the distribution of the marker protein in the LAT-ISH- neurons (Table 4, Fisher’s exact Chi-squared test). For the marker proteins Ret and CGRP, there was no significant difference in the percentage of Marker+ neurons for LAT-ISH+ cells and LAT-ISH- cells. In the case of TrkA, nNOS, RT97 and peripherin, there was a significant difference. Comparison of the expected and observed frequencies in each case suggests that fewer of the LAT-ISH+ cells are also Marker+ than would be expected based on the distribution of the marker in LAT-ISH- cells, and thus that LAT is preferentially expressed in Marker- cells.

## Discussion

The major findings of this study are: 1) neuronal markers can be used to characterize neuronal populations within the human TG, 2) a subgroup of neurons was observed to display an overlap with all tested combinations of markers, with the marker combination Ret/CGRP occurring more frequently than expected by chance, and 3) HSV-1 LAT associates proportionally with the neuronal marker proteins Ret and CGRP, in a divergence from the animal model.

The most prevalent of the six neuronal marker proteins investigated was TrkA, with 61.82% of neurons staining positive. This is higher than the 2.60%, 6.22% and 34.61% of Marker+ neurons seen in three adult human TG from two studies [30], [31], but lower than the 70% reported in rat DRG [32]. The median cross-sectional area of TrkA+ neurons in this study (1035 µm^2^) matches well with previous reports of TrkA neurons in rat and human TG [31], [33]. The presence of nNOS in 33.13% of neurons was also higher than previous work on brain NOS in mouse TG (3% of neurons) [21], suggesting possible differences between species. No data are available in the literature on nNOS frequency in human TG. The percentage of neurons positive for Ret (26.93%) matched previously published results of 23.23% positive neurons from a single adult human TG [34] though this was lower than the 61.9% of positive mouse DRG neurons [35], again highlighting potential differences when comparing species and sensory ganglia. The 39.26% of neurons stained with antibody RT97 are within the range of the 23–41% of positive neurons in mouse TG [21], [36]. No data on staining frequency in the human TG for the RT97 antibody is available. The larger cross-sectional area of these neurons compared to Marker- neurons reflects the association of this antibody with larger A-type neurons [19]. Results with the CGRP antibody were slightly lower than those seen in other work of 30–50% positive TG neurons in mice, rats and humans [20], [37], [38]. Interestingly, the percentage of neurons positive for CGRP also seemed to decrease with increasing donor age, though this was based on a small sample number. Peripherin was found in neurons with a smaller cross-sectional area, reflecting previous work on this marker in rats [39], [40]. Little data is available on frequency in the TG – a recent paper found 72.3% of TG neurons traced from rat upper molars along dental primary afferents to the maxillary region of the TG were peripherin+ [39], but no data are available on frequencies in human TG.

The total percentages of the six neuronal marker protein frequencies suggested that these proteins were overlapping in some neurons. This was confirmed for a subset of antibodies using dual immunofluorescence. Comparison of the dual staining to the proportion of neurons labeled by each antibody in the neuron population as a whole revealed that TrkA/Ret, TrkA/RT97, and Ret/nNOS dual staining occurred less frequently than would be expected. This reflects previous work in the literature that TrkA and Ret are present in two distinct populations of nociceptive neurons [35], and that RT97 is labeling larger A-type neurons in comparison to the smaller B-type neurons that contain TrkA [19]. All of the markers used in the present study stained neurons from a wide range of sizes with a large overlap between the different neuronal markers. The Ret/CGRP dually labeled neurons occurred more frequently than expected by chance. Lectin BSL IB4 (which labels a roughly comparable neuron population to the marker Ret [18]) has been shown to co-label neurons with CGRP in the mouse and more so in the TG of rats [41], [42]. Ret and CGRP both label neurons related to pain signaling, albeit they are commonly considered to be located in different classes of neurons [43].

LAT was found using ISH in neurons expressing all of the six marker proteins studied, reflecting the work in animal models that all neurons are susceptible to HSV-1 infection [21]. It is still a matter of debate whether ISH can detect LAT in all latently infected neurons in the human TG or if some neurons do not express LAT to a level that allows for detection using this method, and thus neurons expressing LAT at a very low level could have been missed in the present study [27], [28], [44]. The distribution of LAT in Ret+ or CGRP+ neurons reflected the proportion of these neurons in the population, whilst LAT was found less often in neurons labeled with antibodies against TrkA, nNOS, RT97 and peripherin. None of the marker proteins investigated showed a positive correlation between LAT and the marker. This suggests either that LAT has a preference for a certain neuron type that was not identified by one of the six markers in this study, or alternatively that there is a neuron population characterized by a combination of two or more of the marker proteins from this study. The statistically significant co-localization of Ret and CGRP in a subset of neurons suggests that these might be a target, a finding that warrants further investigation.

The reduced association of LAT with RT97+ and peripherin+ neurons could reflect a preference for intermediate sized B-type neurons, as RT97 in this study and the literature [45] labels larger neurons, whilst peripherin was found in neurons with a smaller cross-sectional area. In fact, the average size of LAT-ISH+ neurons best fitted with the size of neurons stained with antibody against Ret, which did not show this negative distribution. The reduced association of LAT with nNOS+ neurons in this study could also reflect the potential anti-viral activity of NO seen in animal models [21], or the association of nNOS+ neurons with the ophthalmic branch of the TG, which shows less latent infection in humans [20], [46]. LAT was found in CGRP neurons as would be expected based on the magnitude of this population. If these neurons are associated with innervation of the gingiva as is seen in rats [33] then this could be important based on the oral route of human HSV-1 infection, but the distribution of CGRP in the branches of the human TG has not so far been investigated.

## Conclusions

In the present study co-localization of LAT using ISH occurred most frequently with neuronal markers Ret or CGRP, two markers which also preferably co-localize within the same neuron population. The distribution of LAT in the human TG in this study contrasts with the mouse model, where A5+ neurons, which have a high co-localization with TrkA+ neurons, are more permissive for HSV-1 latent infection, whilst KH10+ neurons, which have a high co-localization with Ret+ neurons, are less permissive [18], [21], [23]. The disparity between the mouse and human results is also intriguing in light of the failure of the mouse model to show full spontaneous reactivation similar to that seen in humans and rabbits [47]–[49]. This difference could be purely due to inherent differences in the distribution of these neuronal markers between species or related to other host factors such as immunological factors. It could also reflect the ocular route of infection used in the mouse model, as compared to the typical route of human infection via the oral mucosa, as different neuronal populations could innervate these tissues differentially [50]. However, in the rabbit model, the ocular route of infection is also often used [49]. Since the clinical presentation of HSV-1 reactivation can result in differential diseases, among them herpes labialis, facial palsy, vestibular neuritis or encephalitis, one might speculate that from the clinical localization and distribution of HSV-1 reactivation, one could infer the affected neuronal subpopulation.

## Supporting Information

Figure S1 **Labeling of a TG with isotypes of all markers used in the current study**. Isotype control for mouse primary antibody (08-6599), Life Technologies, Darmstadt, Germany; Isotype control for rabbit primary antibody (086199), Life Technologies; Isotype control for goat primary antibody (02-6202), Life Technologies; Isotype control for sheep primary antibody (013-000-002), Jackson Immuno Research Europe Ltd., Suffolk, UK. The micrograph on the left was taken for an overview, that on the right for a more detailed view. The tissue was counterstained with haematoxylin. Scale bars represent 50 µm in all cases.
(TIF)

Figure S2 **Labeling of TG with isotypes for each marker used in the current study.** Isotype control for mouse primary antibody (08-6599), Life Technologies, Darmstadt, Germany; Isotype control for rabbit primary antibody (086199), Life Technologies; Isotype control for goat primary antibody (02-6202), Life Technologies; Isotype control for sheep primary antibody (013-000-002), Jackson Immuno Research Europe Ltd., Suffolk, UK. The name of the isotype used is indicated in each row. The micrograph on the left was taken for an overview, that on the right for a more detailed view. The tissue was counterstained with haematoxylin. Scale bars represent 50 µm in all cases.
(TIF)

Figure S3 **Fluorescence co-labeling of isotype controls.** The isotypes are labeled with Alexa568 (red), Alexa488 (green) and DAPI (blue). Isotype combinations are indicated above each pictogram. The letter “U” indicates examples of unlabeled neurons, some with lipofuscin. Scale bars represent 50 µm.
(TIF)

Table S1 **The median and interquartile ranges of Marker+ neurons using immunofluorescence.**
(DOCX)

Table S2 **The median and interquartile ranges of LAT-ISH+ and Marker+ neurons.**
(DOCX)
