# Supplementary figures and images for: Characterization of Neuronal Populations in the Human Trigeminal Ganglion and Their Association with Latent Herpes Simplex Virus-1 Infection
Source: PLoS One. 2013 Dec 19;8(12):e83603. doi: 10.1371/journal.pone.0083603 (PMC3868591; doi:10.1371/journal.pone.0083603)

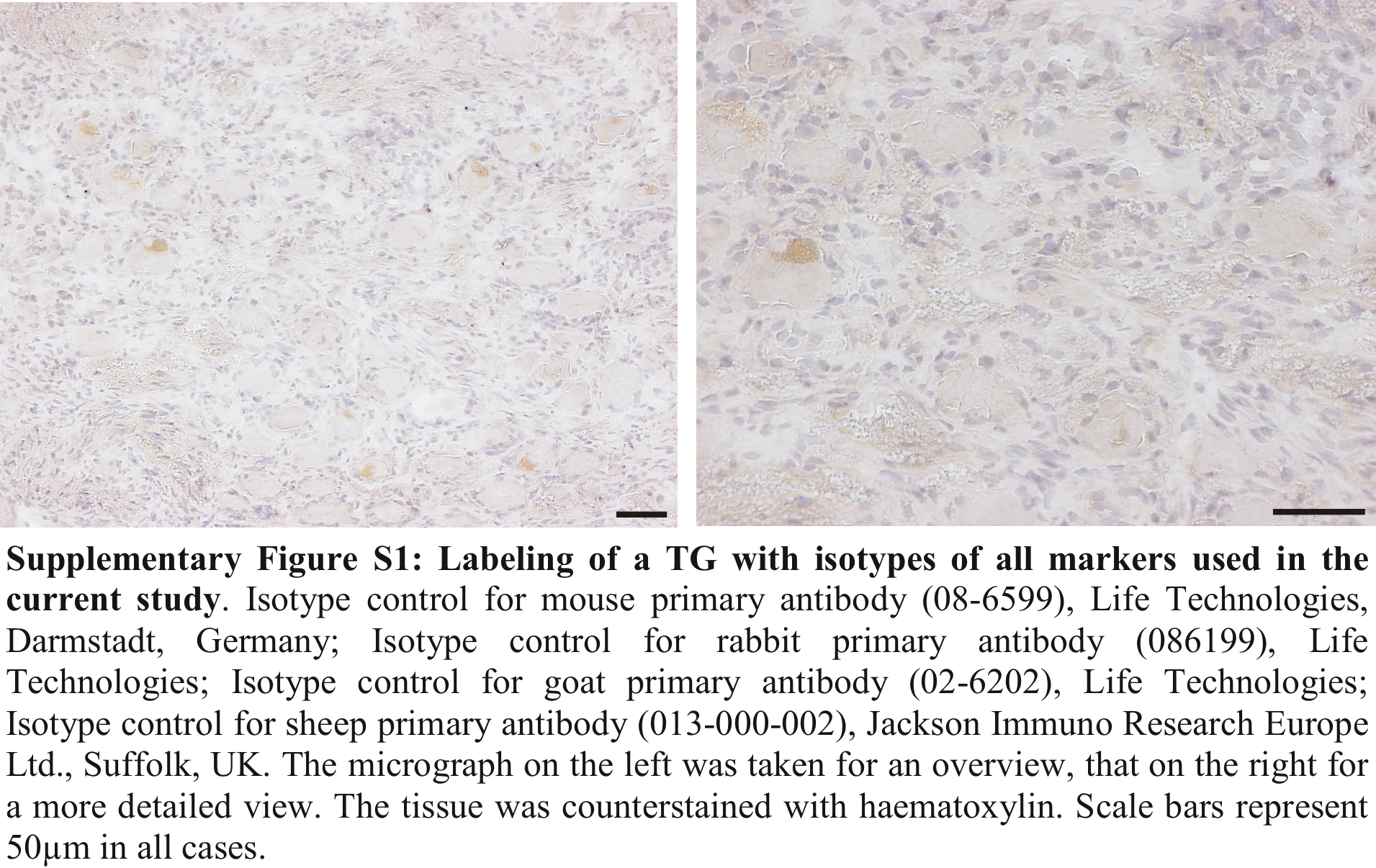

Supplement: Figure S1 — Labeling of a TG with isotypes of all markers used in the current study. Isotype control for mouse primary antibody (08-6599), Life Technologies, Darmstadt, Germany; Isotype control for rabbit primary antibody (086199), Life Technologies; Isotype control for goat primary antibody (02-6202), Life Technologies; Isotype control for sheep primary antibody (013-000-002), Jackson Immuno Research Europe Ltd., Suffolk, UK. The micrograph on the left was taken for an overview, that on the right for a more detailed view. The tissue was counterstained with haematoxylin. Scale bars represent 50 µm in all cases. (TIF) [file pone.0083603.s001.tif]

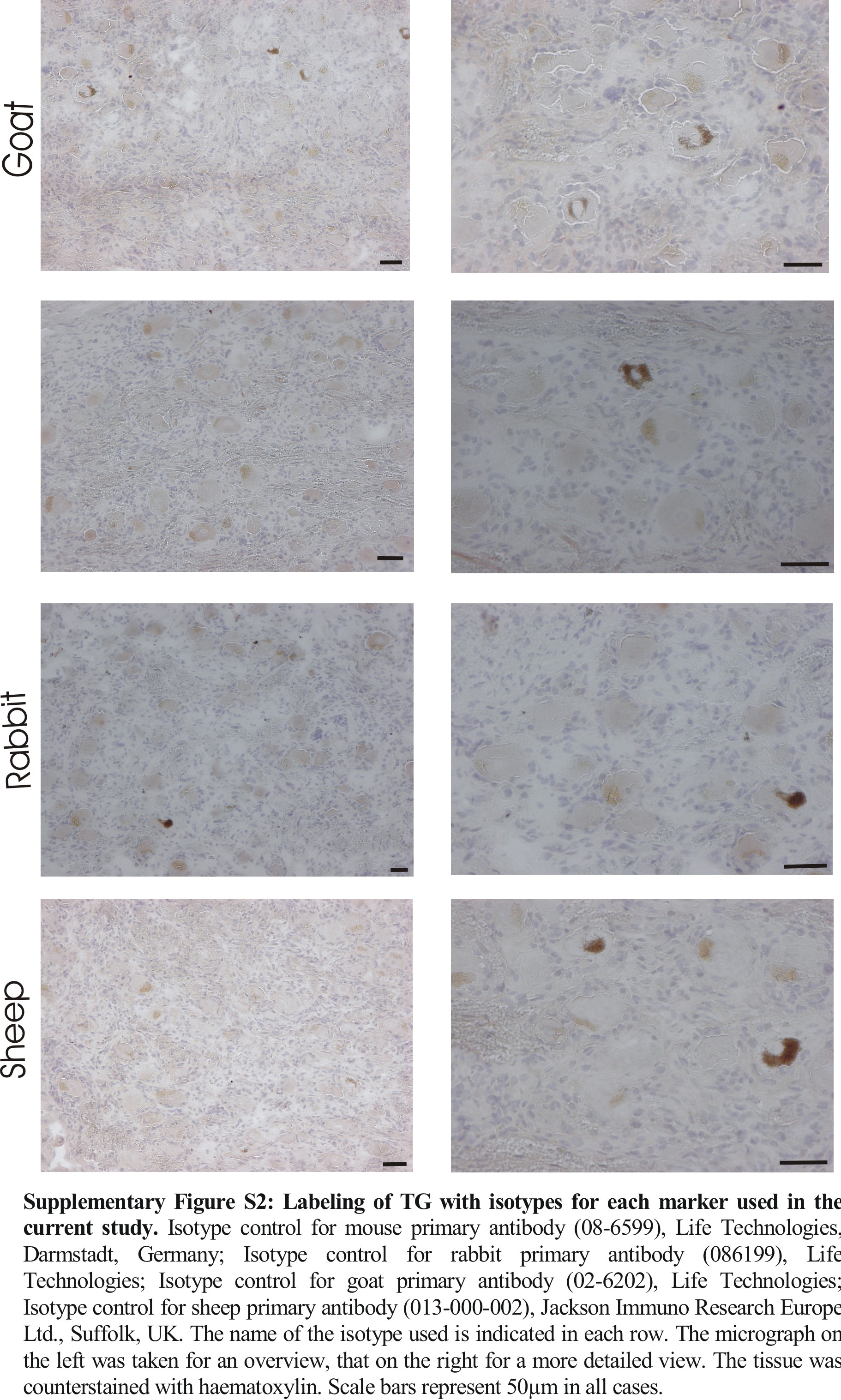

Supplement: Figure S2 — Labeling of TG with isotypes for each marker used in the current study. Isotype control for mouse primary antibody (08-6599), Life Technologies, Darmstadt, Germany; Isotype control for rabbit primary antibody (086199), Life Technologies; Isotype control for goat primary antibody (02-6202), Life Technologies; Isotype control for sheep primary antibody (013-000-002), Jackson Immuno Research Europe Ltd., Suffolk, UK. The name of the isotype used is indicated in each row. The micrograph on the left was taken for an overview, that on the right for a more detailed view. The tissue was counterstained with haematoxylin. Scale bars represent 50 µm in all cases. (TIF) [file pone.0083603.s002.tif]

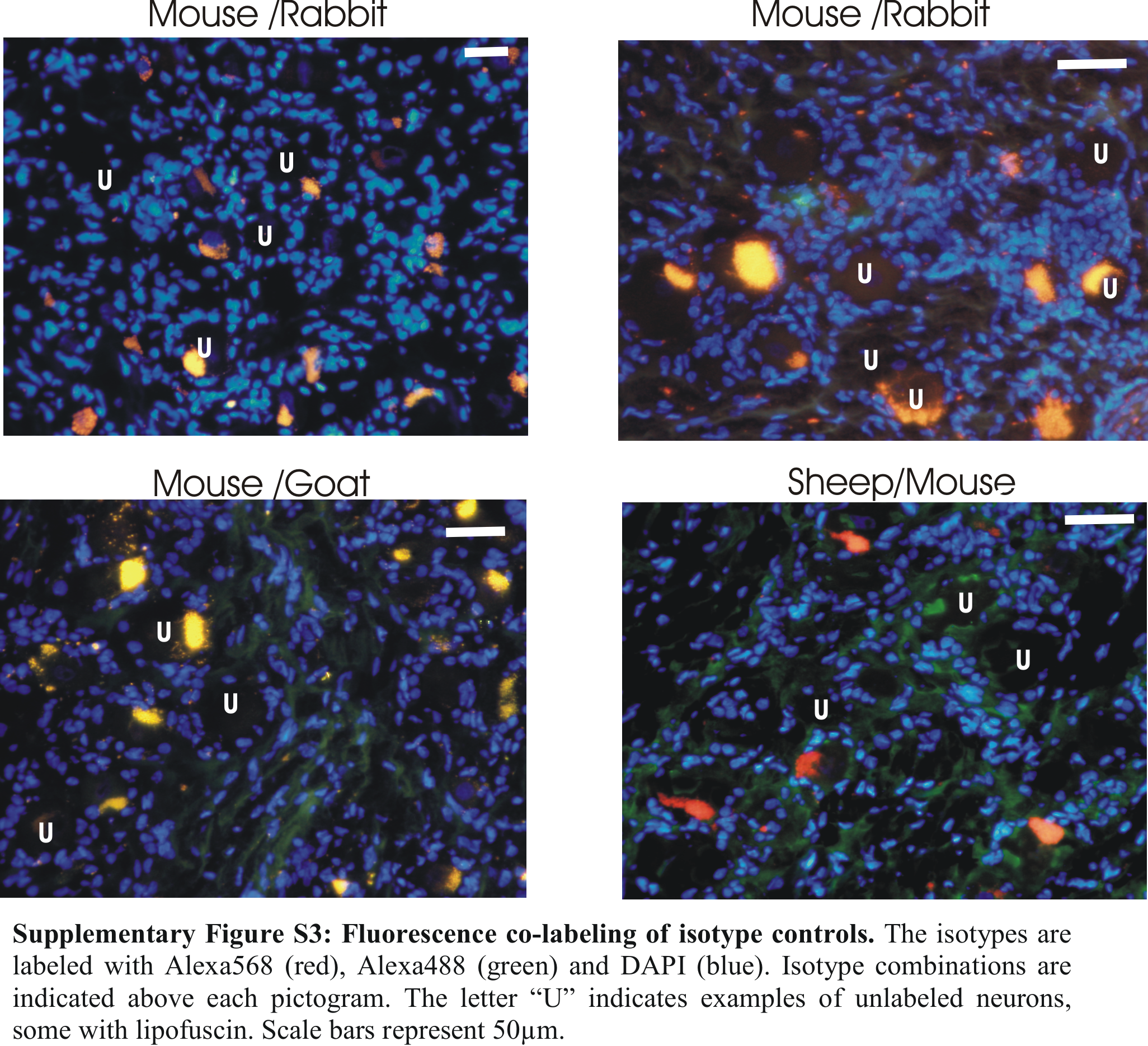

Supplement: Figure S3 — Fluorescence co-labeling of isotype controls. The isotypes are labeled with Alexa568 (red), Alexa488 (green) and DAPI (blue). Isotype combinations are indicated above each pictogram. The letter “U” indicates examples of unlabeled neurons, some with lipofuscin. Scale bars represent 50 µm. (TIF) [file pone.0083603.s003.tif]
