# Supplementary material for: Characterization of Neuronal Populations in the Human Trigeminal Ganglion and Their Association with Latent Herpes Simplex Virus-1 Infection
Source: PLoS One. 2013 Dec 19;8(12):e83603. doi: 10.1371/journal.pone.0083603 (PMC3868591; doi:10.1371/journal.pone.0083603)
Supplement: Table S1 — The median and interquartile ranges of Marker+ neurons using immunofluorescence. (DOCX) [file pone.0083603.s004.docx]

| Marker 1 | Marker 2 | Marker 1+ Median (25th, 75th percentiles) | Marker 2+ Median, (25^th^, 75th percentiles) |
| --- | --- | --- | --- |
| Ret | nNOS | 27 (25,28) | 20 (19, 24.25) |
| Ret | CGRP | 31.5 (29.75, 38) | 30.5 (29, 32.25) |
| TrkA | Ret | 42 (39.25, 47) | 24.5 (20.75, 26) |
| TrkA | nNOS | 46.5 (43.75, 47) | 25.5 (23.5, 28) |
| TrkA | RT97 | 45.5 (44.5, 51.5) | 41 (38.75, 42.5) |
| nNOS | RT97 | 28 (26.25, 28) | 36.5 (32.5, 38.25) |

**Supplementary Table S4: The median and interquartile ranges of Marker+ neurons using immunofluorescence**^a^

a: The medians and percentiles are shown as percentages, indicating the range of values obtained from the various donors in the experiments presented in Table 3 of the main text.
