# Supplementary material for: Characterization of Neuronal Populations in the Human Trigeminal Ganglion and Their Association with Latent Herpes Simplex Virus-1 Infection
Source: PLoS One. 2013 Dec 19;8(12):e83603. doi: 10.1371/journal.pone.0083603 (PMC3868591; doi:10.1371/journal.pone.0083603)
Supplement: Table S2 — The median and interquartile ranges of LAT-ISH+ and Marker+ neurons. (DOCX) [file pone.0083603.s005.docx]

**Supplementary Table S5: The median and interquartile ranges of LAT-ISH+ and Marker+ neurons**^a^

| Marker experiment | LAT-ISH+ Median (25th, 75th percentiles) | Marker+ Median (25th, 75th percentiles) |
| --- | --- | --- |
| Ret | 4.93 (2.92, 5.31) | 24.22 (22.12, 27.05) |
| TrkA | 5.39 (4.37, 5.65) | 58.33 (58.09, 58.87) |
| nNOS | 5.58 (4.74, 5.84) | 34.20 (29.56, 36.69) |
| RT97 | 3.24 (2.70, 3.62) | 40.27 (39.04, 43.50) |
| CGRP | 3.77 (3.13, 3.79) | 26.77 (23.57, 27.18) |
| Peripherin | 3.65 (3.35, 3.88) | 39.38 (38.32, 44.75) |

a: The medians and percentiles are shown as percentages, indicating the range of values obtained from the various donors in the experiments presented in Table 4 of the main text.
